# 2300. Incidence of COVID-19 in Irish Healthcare Workers: Preliminary Results from PRECISE-5

**DOI:** 10.1093/ofid/ofad500.1922

**Published:** 2023-11-27

**Authors:** Claire Kenny, Jonathan McGrath, Gavin Kelly, Shane Walsh, Conor Moran, Lee Flynn, David Byrne, Irene Flynn-Dowling, Niamh Allen, Peadar Rooney, Greg Martin, Lorraine Doherty, Catherine Fleming, Colm Bergin

**Affiliations:** Galway University Hospital, Galway, Galway, Ireland; St. James's Hospital, Dublin, Dublin, Ireland; Galway University Hospital, Galway, Galway, Ireland; St. James's Hospital, Dublin, Dublin, Ireland; St. James's Hospital, Dublin, Dublin, Ireland; St. James's Hospital, Dublin, Dublin, Ireland; St. James's Hospital, Dublin, Dublin, Ireland; St. James's Hospital, Dublin, Dublin, Ireland; St. James's Hospital, Dublin, Dublin, Ireland; Galway University Hospital, Galway, Galway, Ireland; Health Protection Surveillance Centre (HPSC), Dublin, Dublin, Ireland; Health Protection Surveillance Centre (HPSC), Dublin, Dublin, Ireland; Galway University Hospital, Galway, Galway, Ireland; St. James Hospital, Dublin, Dublin, Ireland

## Abstract

**Background:**

Healthcare workers (HCW) are the ideal population to track the evolving epidemiology of SARS-CoV-2. Surges in breakthrough SARS-CoV-2 infection and quarantine regulations leads to staff absence at a time when hospital resources are already burdened.

**Methods:**

All HCWs in 2 large teaching hospitals were invited to participate in this prospective longitudinal cohort study in November 2022. Information on demographics, SARS-CoV-2 infection history and COVID-19 vaccination dates/brands was collected. Baseline serology for anti-Nucleocapsid (N) and anti-Spike (S) antibodies was performed. Cohort seroprevalence was compared to a prior study phase (November 2021). Monthly follow-up e-surveys collected information relating to incident SARS-CoV-2 infections, symptom duration and work days missed.

**Results:**

1261 participants enrolled (Table 1). 25/1261 (0.08%) were unvaccinated. 1103/1261 (87.5%) had received a primary vaccine series and at least one booster.

1260/1261 (99.9%) of participants were anti-S seropositive. 1008/1261 (79.9%) participants were anti-N seropositive. In comparison, serology performed in November 2021 showed anti-N antibody seropositivity in 23.4% (p< 0.0001). 377/1261 (29.9%) participants denied having a prior SARS-CoV-2 infection; of these, 178/377 (47.21%) were anti-N seropositive suggestive of previously undiagnosed infection.

Response rate to monthly surveys over the first 14 weeks of the study (Weeks 45-52 of 2022 and Weeks 01-06 of 2023) was 54.3%. 121/1261 (9.6%) of participants reported a positive SARS-CoV-2 test (PCR or Antigen). Incident infections peaked at Weeks 50-52 of 2022 (Fig. 1), reflective of the national trend. 41/121 (33.8%) reported a symptomatic infection, with median symptom duration 7.5 days. Over 14 weeks, total number of work days missed secondary to SARS-CoV-2 infection was 589 days (median 5 days missed per HCW).

**Figure:**
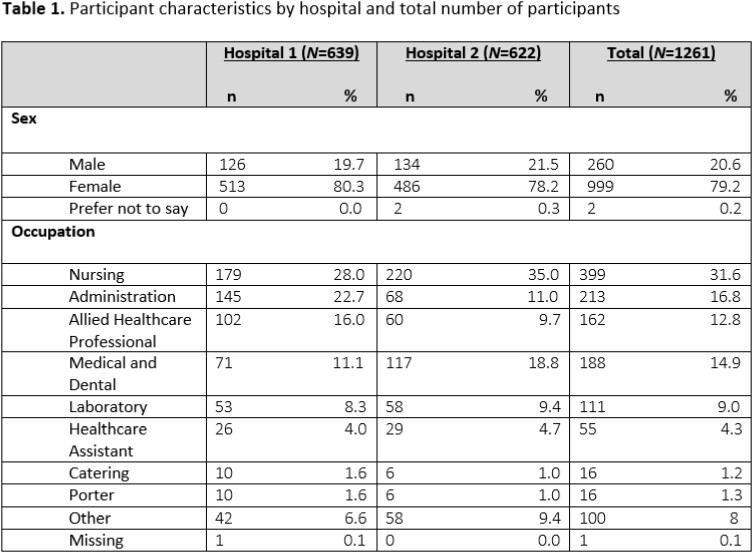

**Figure:**
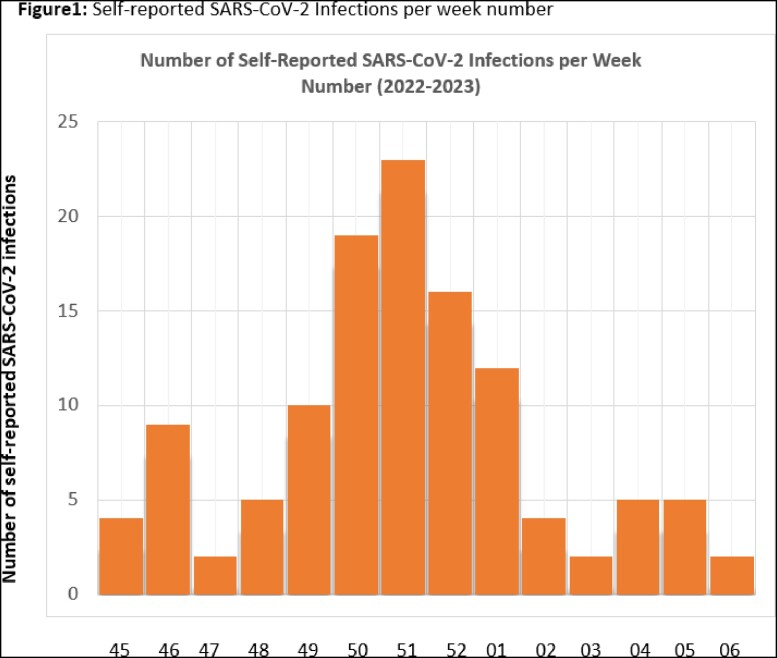

**Conclusion:**

In this interim analysis, 9.6% of participants reported an incident SARS-CoV-2 infection over 14 weeks. This was in the context of high baseline seropositivity for anti-N antibody and vaccination uptake. This suggests that SARS-CoV-2 continues to circulate widely. While morbidity and mortality is low, there are significant consequences including rostered working days lost in healthcare settings.

**Disclosures:**

**All Authors**: No reported disclosures

